# On the Suitability of Almond Shells for the Manufacture of a Natural Low-Cost Bioadsorbent to Remove Brilliant Green: Kinetics and Equilibrium Isotherms Study

**DOI:** 10.1155/2021/6659902

**Published:** 2021-01-29

**Authors:** R. Melhaoui, Y. Miyah, S. Kodad, N. Houmy, M. Addi, M. Abid, A. Mihamou, H. Serghini-Caid, S. Lairini, N. Tijani, C. Hano, A. Elamrani

**Affiliations:** ^1^Laboratoire dʼAmélioration des Productions Agricoles, Biotechnologie et Environnement, (LAPABE), Faculté des Sciences, Université Mohammed Premier, Oujda, Morocco; ^2^Laboratoire de Catalyse, Matériaux et Environment, EST, Université Sidi Mohammed Ben Abdellah, Fez, Morocco; ^3^Equipe de Recherche, Membranes, Matériaux et Procédés de Séparation, Faculté des Sciences, Université Moulay Ismaîl, Meknès, Morocco; ^4^Laboratoire de Biologie des Ligneux et des Grandes Cultures, INRAE USC1328, University of Orleans, Orleans, France

## Abstract

Almond production generates a large number of coproducts, but the farmer's interest mainly focuses on the nutritional and commercial aspects of the kernel for getting the best return from their harvests. Thus, almond coproducts such as almond shells that represent more than 70% of biomass remain underexplored. In this work, the suitability of almond shell powder (ASP) as a natural low-cost adsorbent was evaluated in the adsorption of brilliant green dye (BG), which is known as a chemical pollutant. Brunauer–Emmett–Teller (BET) method, for the determination of specific surface area, Fourier-transform infrared spectroscopy (FTIR), and scanning electron microscopy (SEM) techniques were performed to characterize the ASP adsorbent. The batch adsorption kinetic study for the removal of BG dye was carried out by varying pH, temperature, initial concentration of the dye, bioadsorbent dose, and contact time. It was found that 98% of BG dye is removed under the following optimal experimental conditions: ASP bioadsorbent dose of 1 g/L at *T* = 25°C, pH = 6.8, and *C*_0_ = 1 g/L, which proves that ASP can be used as an excellent low-cost bioadsorbent for the removal of BG dye from wastewater. The experimental isotherm data were analyzed using Freundlich and Langmuir models. The results show the best correlation with single-layer adsorption, and the adsorption kinetics seems to follow a pseudo-second-order model.

## 1. Introduction

Industrial pollution is a major factor causing the degradation of the environment. Water pollution caused by industrial effluents threatens biological life in aquatic systems; its impact on human health is no longer to be demonstrated and becomes a worrying problem to consider before it is too late. Wastewater contamination with harmful dyes is a serious matter because their low biodegradability, high toxicity, and dyes residues cause many health problems [1, 2]. Dyes are used by many industries like textile, leather, food, cosmetics, and plastic, as a coloring agent. In recent decades, the uses of dyes seem to continue increasing in different sectors particularly in the textile [3] and the food industry [4]. This increase is mainly due to their facility and the fastness of the synthesis, along with their economic interest [5]. For instance, the textile industry produces colored toxic effluents, which are generally resistant to destruction by biological treatment methods [6]. Among them, brilliant green (BG) is a typical example of toxic cationic dye extensively used in textile dyeing and paper printing [7], with harmful effects on humans. It may cause dermatitis upon skin contact and irritations to both the gastrointestinal and respiratory tracts in humans, resulting in a cough and shortness of breath [8, 9]. Indeed, colored wastewater causes environmental problems along with serious damage to the aquatic ecosystem and groundwater. Several treatments, including biological and physiochemical treatments, have been developed [7, 8, 10–13]; however, some of them face certain technical and economical limitations. Therefore, adsorption with low-cost adsorbents seems to be an effective and economic method for industrial effluent treatment. The most used adsorbents are clay-based materials because of their environmental-friendly nature materials [14]. Also, several other natural adsorbents of plant origin, such as coconut [15, 16], date stones [17], and orange and potato peels [18–20], have been studied and optimized. This research focuses on the suitability of almond shells for the manufacture of an eco-friendly low-cost bioadsorbent to remove dyes from industrial effluents. Indeed, in eastern Morocco, many farm cooperatives are gathered into economic interest groups (EIGs). They are faced with the valorization challenges of almonds and their coproducts. Their aim is to empower smallholders to reach their potential and encourage reliable growth for their businesses, communities, and livelihoods. Besides kernels, they seek to enhance the value of almond coproducts and hulls to feed livestock, but also shells, which are generally underestimated and burned as a source of energy, and that will be used in this study for the manufacture of a low-cost organic adsorbent for the removal of dye from industrial colored effluents in wastewater treatment.

## 2. Materials and Methods

### 2.1. Preparation of Adsorbate

Brilliant green (BG dye) is a cationic dye supplied by Riedel-de-Haën and used for various purposes. Physicochemical characteristics of BG are shown in Table 1. A dye stock solution of 1 g/L was prepared at ambient temperature by dissolving BG in distilled water. The homogenization of the solution was ensured by a magnetic stirrer.

### 2.2. Description and Preparation of the Bioadsorbent

ASP that was used in this work comes from two French varieties, *Ferragnes* and *Ferraduel*, which are the most cultivated in the eastern region of France. The first step in the preparation of the ASP sample was the collection of the almond in the different study orchards. The second step was to shell the almond fruits, which are then washed several times with distilled water until pH becomes stable in order to remove dust and other inorganic impurities. The obtained shell was dried in the oven for 24 hours at 60°C.

**Figure 1:**
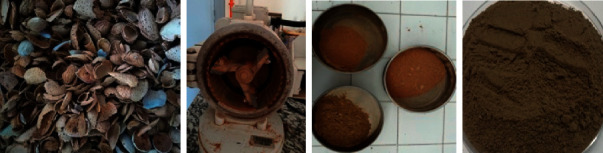
The followed steps in the preparation of adsorbent powder from almond shells: almond shells' grinding, sieving, and size grading. Almond shell powder (ASP) for adsorption essays.

The samples were ground and sieved to get the particles with a size less than 400 *μ*m.  Figure 1 illustrates the essential steps followed to prepare the ASP samples.

### 2.3. Experimental Adsorption Procedure

The characterization of almond shell powder (ASP) was done with FTIR (Fourier Transform Infrared spectroscopy, BRUKER, Vertex70), in the 400–4000 cm^−1^ wavenumber range. The morphology was observed using a scanning electron microscope (Quanta200 FEI equipped with EDX probe for surface microanalysis). The texture of the ASP powder (specific surface area and pore volume) was determined by the Brunauer–Emmett–Teller (BET) and the Barret–Joyner–Halenda (BJH) methods [21], which are based on N_2_ adsorption/desorption isotherms performed at 77°K on a micromeritics apparatus (ASAP 2010). Before each analysis, the sample is degassed at *T* = 80°C to remove the physisorbed impurities.

The value of pH of zero point charge (pHzpc) for ASP is determined by the electrochemical method, at different precise initial pHi values between 2 and 12 by the addition of NaOH and HCl 0.1 Mol/L. Then, 0.5 g of adsorbent was added to each beaker; after stirring for 48 h, the final pHf was measured. The value of pHzpc is determined by the intersection point of the curves carrying ΔpH = pHf − pHi as a function of pHi [22].

Adsorption tests of BG dye onto the ASP sample were accomplished in a glass beaker of 500 mL capacity at ambient temperature *T* = 25°C. Different stock solutions of BG (30–50 mg/L) and adsorbent dose (1-2 g/L) are prepared using distilled water to carry out kinetics and isotherms of BG dye adsorption. The homogenization of the solutions was ensured by a magnetic stirrer. After each 5 min of contact time *t*, the solution was filtered using a syringe filter with 0.45 *μ*m diameter (Minisart, Sartorius Stedim Biotech). The effects of different variables such as the concentration of dye (30–40 and 50 mg/L), the ASP amount (1-1.5-2 g/L), the contact time (0–60 min), pH of the solution (adjusted in the range of 4 to 10 by the addition of HCl and NaOH (0.1 mol/L), and the temperature of the solution (in the range of 20 to 50°C) are studied. To determine the dye concentrations, after each adsorption test, the calibration curves of known concentrations (C) of dye were established, according to Beer's law: *A* = *ε*·*l*·*C* (*ε*: molar absorptivity and *l*: length of cuvette) [23] at a wavelength of 625 nm using the UV-Visible spectrophotometer (Jasco V530).

The amount of equilibrium adsorption *q*_*e*_ (mg/g) and the percentage of a dye removal efficiency % (*R*) were calculated using the following equations [24–26]:

(1) $$\begin{array}{c} q_{e}=\frac{C_{0}-C_{e}}{m}\cdot V, \end{array}$$

(2) $$\begin{array}{c} \%R=\frac{C_{0}-C_{e}}{C_{0}}\cdot 100, \end{array}$$

where *C*_0_ (mg/L) is the initial dye concentration, *C*_*e*_ (mg/L) is the concentration at equilibrium adsorption, and *V* and *m* are the volume of the solution (*L*) and the amount of adsorbent (*g*), respectively.

## 3. Results and Discussion

### 3.1. Structural and Surface Characterization of the Adsorbent

**Figure 2:**
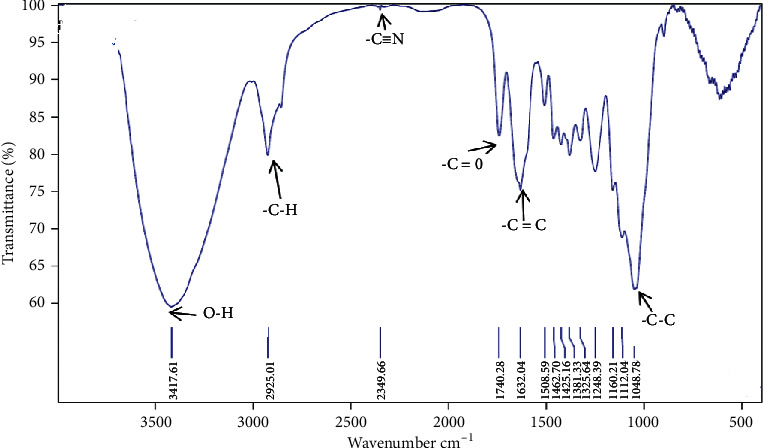
FTIR spectrum of the analyzed almond shell powder (ASP).

The first characterization of ASP is realized by Fourier transform infrared spectroscopy, in the 400–4000 cm^−1^ wavenumber range. The obtained spectrum (Figure 2) shows several absorption bands characterizing the analyzed almond shell powder sample. The band located at 3417 cm^−1^ represents the OH stretching vibrations [27], and the band at 2925 cm^−1^ corresponds to C–H stretching vibrations in the aliphatic chain [28]. The three bands at 1425 cm^−1^, 1381 cm^−1^, and 1325 cm^−1^ correspond to CH_2_ bending vibrations, C–H asymmetric deformation, and CH_2_ wagging, respectively [29]. The band at 1740 cm^−1^ corresponds to the elongation of the C=O bond attributed to aldehydes or saturated acid [12]. The bands at 1632 cm^−1^ and 1508 cm^−1^ are located around the frequency of stretching vibrations of a double bond in the alkene (-C=C) and (C-C) band vibrations that appear at 1048 cm^−1^ [10, 30].

The textural characteristics of ASP determined by the BET method are summarized in Table 2.

**Figure 3:**
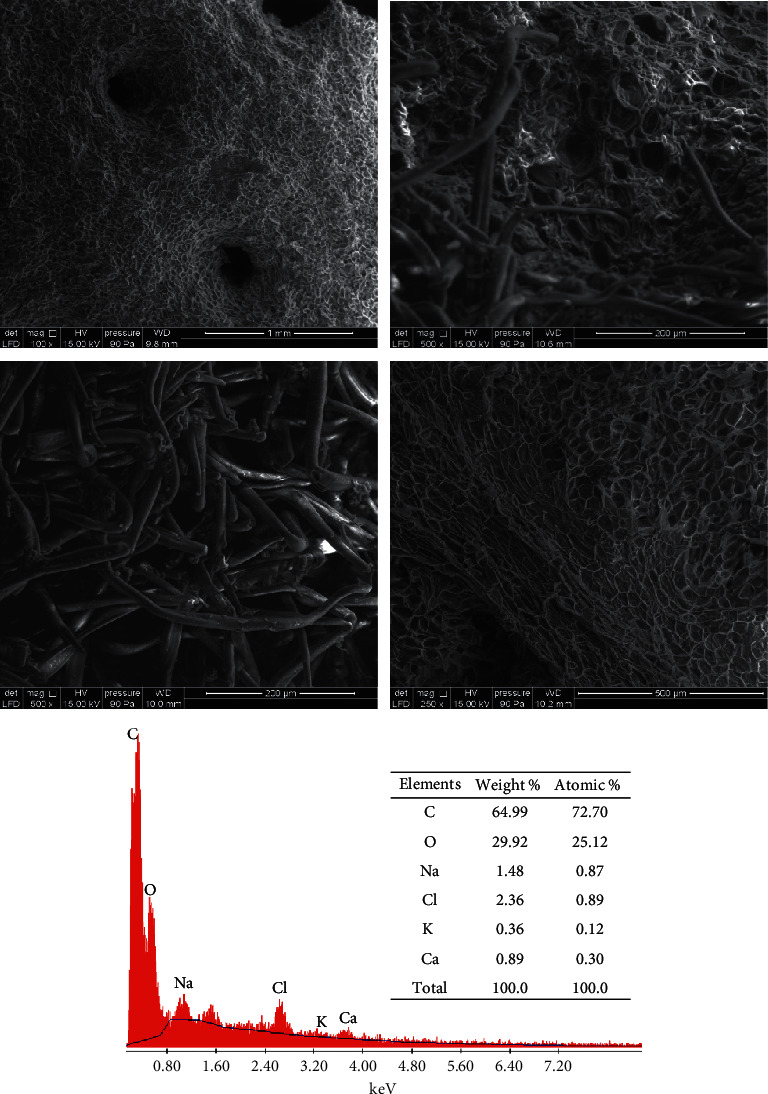
SEM micrographs coupled by EDX of almond shell powder (ASP) at different magnifications, showing surface morphology and porous structure.

These results show a porous structure of the analyzed ASP sample. The scanning electron microscopy (SEM) confirms this result and allows us to characterize the surface morphology of the ASP as seen in Figure 3, which indicates that the ASP sample was composed of irregular pores. The images obtained show also cellulose fiber of various forms and sizes, similar to those observed by SEM analysis of cellulose extracted from almond ASP [31]]. These results show that the morphology of ASP is characterized by a high porosity, which may provide more contact sites to adsorb the synthetic dyes and heavy metals.

The EDX spectrum shown in Figure 3 confirms the presence of large percentages of carbon and oxygen which are in the order of 64.99 and 29.92%, respectively, which confirms the organic nature of the adsorbent material. This chemical analysis by EDX also gives an idea of the nature of the atoms in our adsorbent, which makes it possible to validate the presence of these elements by other characterization techniques.

### 3.2. Effects of Adsorbent Dose

**Figure 4:**
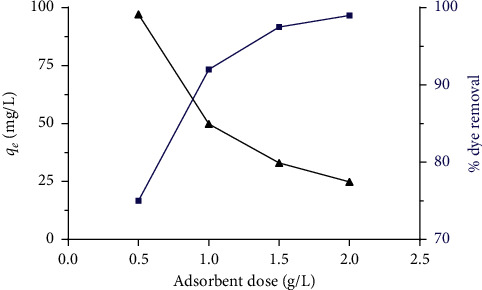
Percentage removal efficiency of BG and the amount adsorbed as a function of the adsorbent dose. Adsorbent amount range: 0.5–2 g/L, pH: 6.8, initial dye concentration of dye: 50 mg/L, *V* = 200 mL, *T*: 25°C, and contact time: 15 min.

The effects of the different adsorbent amounts of ASP (*m* = 0.5, 1, 1.5, and 2 g/L) in the adsorption of an initial concentration of a BG solution of 50 mg/L are presented in Figure 4. The obtained adsorption capacities of ASP are 97.21, 49.85, 33.07, and 24.88 mg/L, respectively. It can be shown that the percentage of dye elimination increases from 75% to 98% when the amount of the adsorbent increases from 0.5 g/L to 2 g/L. This increase could be attributed to the increase of the adsorption site numbers available on the adsorbent surface, in good agreement with other authors [32–34].

### 3.3. Effect of Contact Time and Brilliant Green Concentration

**Figure 5:**
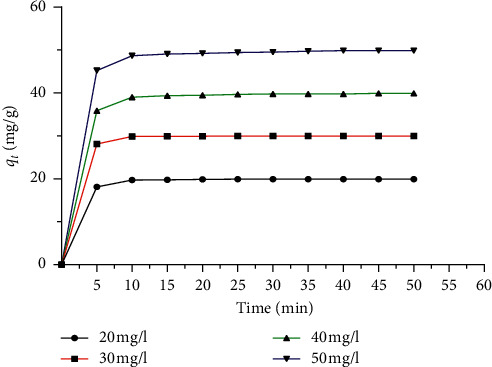
Effect of contact time and the initial concentration of BG on the adsorption capacities of ASP. Interval of initial concentration: 20–50 mg/L, pH: 6.8, adsorbent amount: *W*: 0.2 g, *V*: 200 mL, and *T*: 25°C.

The effect of the concentration in the removal of BG dye at ambient temperature (*T* = 25°C) was studied by taking different initial concentrations ranging from 20 to 50 mg/L and the adsorbent dose of ASP of 1 g/L. The corresponding curves of the adsorption capacity *q*_*e*_ (mg/g) as a function of contact time are represented in Figure 5. The purpose of this study is to determine the contact time, which is considered a very important industrial parameter for the removal of pollutants. It can be observed that the contact time is independent of the initial concentration of BG. The adsorbed quantity of BG increases rapidly in the first 10 min of contact time and remains constant after 15 min indicating an equilibrium state, after which the maximum adsorption capacity of 49.8 mg/L (99% sorption efficiency) was obtained. The rapid adsorption in the initial step (<15 min) indicates that the BG molecules interact easily with the available adsorption sites [35]. However, after this period (>15 min), the rate of adsorption stabilized because of slower diffusion and the saturation of the pores by the BG molecules that resist further adsorption of BG dye [9].

### 3.4. Effect of pH Solution

**Figure 6:**
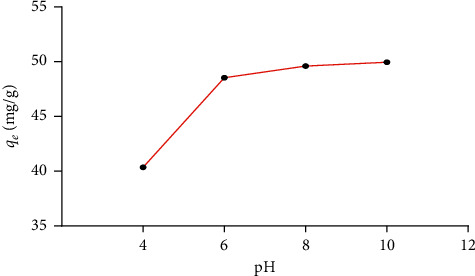
Effect of pH solution in the removal of BG dye. pH range: 4–10, adsorbent amount: 0.2 g, initial dye concentration: 50 mg/L, *V*: 200 mL, and *T*: 25°C.

Initial pH is the most critical parameter affecting and determining dye adsorption [36]. The variation of this parameter influences directly the functional group of adsorbate and the surface charge [37]. In this experience, the pH values ranged from 4 to 10, using an initial concentration of BG of 50 mg/L and 1 g/L of the adsorbent amount.  Figure 6 indicates that the removal of BG increases rapidly from 40.35 to 49.60 mg/g as the pH value increases from 4 to 10. However, pHpzc = 4.51. At pH > 4.51, the surface charge is negative and the dye charge is cationic, which explains the increases in the removal of BG when the pH value is greater than pHzpc = 4.51, due to the electrostatic attraction between cationic dye molecules and the negative surface charge of the ASP adsorbent.

### 3.5. Effect of Temperature

**Figure 7:**
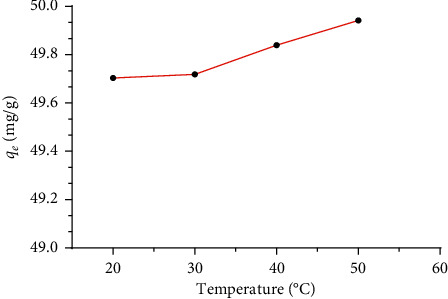
Effect of temperature on adsorption of BG dye onto ASP. Adsorbent amount: 0.2 g, initial BG dye concentration: 50 mg/L, *V* = 200 ml, and temperature range: 20–50°C.

The adsorption temperature is another important parameter that could affect the dye adsorption. The experiments were carried out by adding 1 g/L of ASP to a BG solution (50 mg/L) at different temperatures ranging from 20 to 50°C.  Figure 7 illustrates the obtained results. It can be noticed that the increase of adsorption temperature has a slight effect on the adsorption of BG dye onto the ASP sample. This result indicates that the adsorption, in this case, is slightly endothermic. Consequently, the adsorption process of BG dye onto ASP can be done at ambient temperature, which is highly beneficial for the industry.

### 3.6. Adsorption Kinetics

In order to evaluate the adsorption process, several kinetic models are used to describe the adsorption kinetics, particularly, the pseudo-first-order model proposed by Lagergren [38] and the pseudo-second-order model. These two models were used to identify the kinetics involved in the adsorption of BG by ASP and which will help to determine the kinetic parameters of each model to choose the model that represents the best adsorption process. At lower concentrations of dyes, the most adequate kinetic models are represented, respectively, by the following equations [39]:

(3) $$\begin{array}{c} \text{ln}\left(q_{e}-q_{t}\right)=\text{ln }q_{e}-\text{k}1\text{t,} \end{array}$$

where *q*_*e*_ is the quantity adsorbed with balance (mg·g^−1^), *q*_*t*_ is the quantity of adsorbate adsorbed with time (min), and *k*_1_ is the constant speed of adsorption of the pseudo-first-order model.

(4) $$\begin{array}{c} \frac{t}{q_{t}}=\frac{1}{K_{2}q_{e}^{2}}+\frac{t}{q_{e}}, \end{array}$$

where *K*_2_ is the constant speed of adsorption of the pseudo-second-order model (g·mg^−1^·min^−1^).

**Figure 8:**
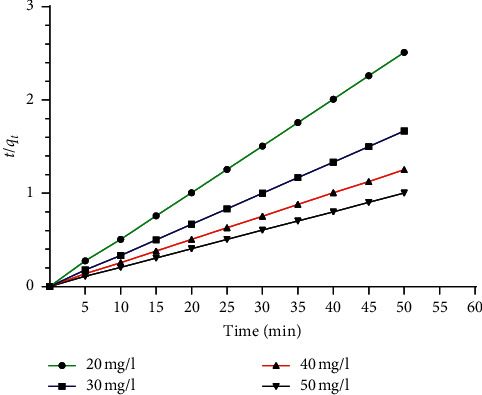
Pseudo-second-order kinetic model for the adsorption of BG onto ASP.

To verify the validity of the models, they can be checked by each linearized plot.  Figure 8 shows that the plot of *t*/*q*_*t*_ versus *t* gives a straight line with a slope of 1/*q*_*e*_ and the *y*-intercept of 1/*k*_2_*q*_*e*_^2^, which is in good agreement with the experimental and the calculated values of *q*_*e*_·exp and *q*_*e*_·cal. The different calculated and experimental parameters (*q*_*e*(cal)_, *K*_1_, *K*_2_, *q*_*e*(exp)_) and the correlation coefficients are given in Table 3. It can be seen that the correlation coefficient for the pseudo-second-order kinetic model (*R*^2^ = 0.99) is greater than that of the pseudo-first-order kinetic model, indicating the validity of this model for the adsorption of BG dye onto the ASP sample.

#### 3.6.1. Intraparticle Diffusion Mechanism

This mechanism is known to occur in three important steps.

- Transport of the adsorbate from the bulk of solution to the ASP surface (boundary layer diffusion)
- Diffusion of the adsorbate into the intraparticle of the solid sample
- Equilibrium where the adsorption slows down due to the lowest concentration of dye in the solution

**Figure 9:**
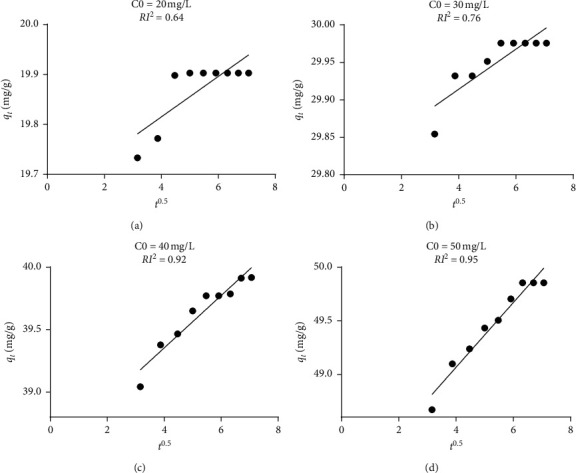
Intraparticle diffusion mechanism for different initial concentrations.

This process is described by the following Weber Morris equation [40]:

(5) $$\begin{array}{c} q_{t}=K_{p}t^{0.5}+I, \end{array}$$

where *K*_*p*_ is the intraparticle rate constant (mg/g min^0.5^), *q*_*t*_ is the amount of solute on the surface of the adsorbent at time *t* (mg/g), *t* is the time (min), and the constant *I* (mg/g) represents the effect of boundary layer thickness. To check this model, the plot of *q*_*t*_ versus *t*^0.5^ should be linear. This is not the case for this model (Figure 9). The linearity of the plots demonstrated that intraparticle diffusion played a significant role in the uptake of BG. It can be observed that when the concentration of dye increases, the multistep process disappears due probably to the increases in the diffusion rate of BG into the pores. However, at a low concentration of dye, the adsorption of GB on the adsorbent was a multistep process, involving adsorption on the external surface and diffusion into the interior. The two phases in the intraparticle diffusion plot suggest that the sorption process proceeds by surface sorption and intraparticle diffusion.

The different calculated and experimental parameters of the intraparticle diffusion model are also mentioned in Table 3.

### 3.7. Adsorption Isotherms of BG Dye onto ASP

To explain the mechanism of the adsorption of BG dye onto ASP samples, the isotherm curves were carried out at different concentrations of dye, using the Langmuir [41] and Freundlich [42] models. The Langmuir isotherm theory is based on the formation of a single layer of adsorbed molecules (forming a molecular monolayer), on each specific site. This model is represented by equation (6), and its linear representation can be rearranged as equation (7).

(6) $$\begin{array}{c} q_{e}=\frac{K_{L}q_{m}C_{e}}{1+K_{L}C_{e}}, \end{array}$$

(7) $$\begin{array}{c} \frac{C_{e}}{q_{e}}=\frac{1}{K_{L}q_{m}}+\frac{C_{e}}{q_{m}}, \end{array}$$

where *K*_*L*_ (L/mg) is Langmuir constant related to the affinity between adsorbate and adsorbent and *q*_*m*_ is the theoretical maximum adsorption capacity at monolayer formation.

To check the favorability of this model, a dimensional parameter called equilibrium parameter *R*_*L*_ defined by equation (8) can be calculated.

(8) $$\begin{array}{c} R_{L}=\frac{1}{1+K_{L}C_{0}}, \end{array}$$

where *C*_0_ (mg/L) is the initial concentration of dye. According to the *R*_L_ value, we allow determining the type of the isotherm, 0 < *R*_*L*_ < 1 (favorable), *R*_*L*_ = 1 (linear), *R*_*L*_ = 0 (irreversible), and *R*_L_ > 1 (unfavorable). The *R*_L_ values ranged from 0.0005 to 0.0012, indicating that the adsorption is a favorable process. In comparison to the Freundlich isotherm, which supposes the heterogeneity of the surface adsorption sites with a multilayer adsorption mechanism, the adsorbed amount increases with adsorbate concentration. The model of Freundlich isotherm described by equation (9) and its transformation into a logarithmic scale equation (10) of this equation makes it possible to check the validity of this model.

(9) $$\begin{array}{c} q_{e}=K_{F}C_{e}^{1/n}, \end{array}$$

(10) $$\begin{array}{c} \text{Ln }q_{e}=\text{Ln }K_{F}+\frac{1}{n}+\text{Ln }C_{e}, \end{array}$$

where *n* is a constant related to adsorption intensity and *K*_*F*_ (mg^1−1/*n*^ g^−1^ L^1/*n*^) is the Freundlich constant characterizing the efficiency of an adsorbent. These parameters of Langmuir and Freundlich constants are mentioned in Table 4.

**Figure 10:**
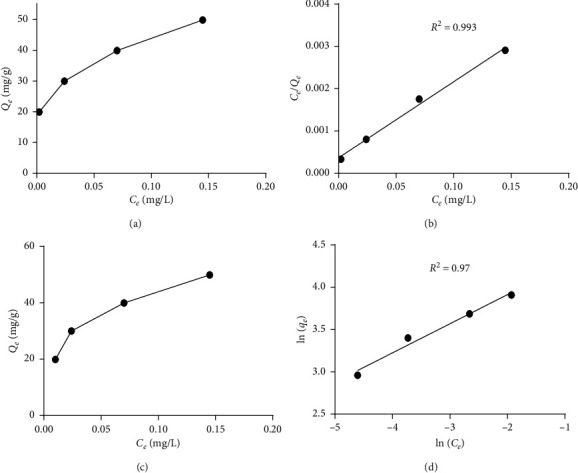
Isotherm model for BG dye adsorption: (a) and (b) Langmuir isotherm model; (c) and (d) Freundlich isotherm model.

According to the correlation coefficient (*R*^2^) of the two isotherms studied (Figure 10), the adsorption of BG dye on almond shell powder **(**ASP) could be well simulated with the isotherm model of Langmuir (*R*^2^ = 0.993) compared to that of Freundlich model (*R*^2^ = 0.97).

Therefore, adsorption of the grinding almond ASP is a physical adsorption type. A comparison of the adsorption capacity of ASP with other alternative biowaste adsorbents is represented in Table 5. The maximum adsorption capacity of ASP calculated from Langmuir isotherm was found to be 58.13 mg/g. Comparing the adsorption capacity of ASP, it is clear that the ASP possesses reasonable adsorption capacity in comparison with other sorbents and can be used to remove BG dyes from wastewater. From the literature survey, it is found that the removal of BG dye using ASP has not been reported by any researcher. This is the first report where ASP is used as an adsorbent. In comparison with other similar works, which used the almond shell as an adsorbent but with other dyes, close results have been found for cationic dyes such as methylene blue and crystal violet with a pseudo-second-order model and Langmuir isotherm. ASP sample shows a high adsorption capacity compared to other adsorbents. To better describe the adsorbate-adsorbent system interaction, we had carried out the kinetic study. We found that the adsorption isotherm is significant to represent the quantitative interaction between molecules of the BG dye and the ASP adsorbent [43]. From the facts, it is clear that our adsorbate-adsorbent system was in good agreement with that of the pseudo-second-order model and Langmuir model. This explains that the adsorption of the dye onto the ASP surface is in monolayer. The results show that the adsorption isotherms of BG on ASP are favorable; similar results have been obtained in other studies for other dyes [44].

### 3.8. Thermodynamics of Adsorption

**Figure 11:**
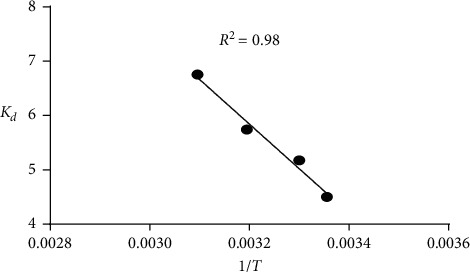
Variation of partition coefficient *K*_*d*_ as a function of 1/*T*. Temperature range: 20–50°C, adsorbent amount (ASP): 0.2 g, initial BG dye concentration: 50 mg/L, and *V*: 200 ml.

The thermodynamic parameters of the adsorption process allow us to determine the feasibility and the favorability of the adsorption. Several parameters are calculated such as entropy change (Δ*S*°), free energy change (Δ*G*°), and enthalpy change (Δ*H*°). The values of Δ*S*° and Δ*H*° were calculated using the equation of Van't Hoff:

(11) $$\begin{array}{c} \text{ln }K_{d}=\frac{\Delta S^{{}^{\circ}}}{R}-\frac{\Delta H^{{}^{\circ}}}{\text{RT}}, \end{array}$$

(12) $$\begin{array}{c} \Delta G^{{}^{\circ}}=\Delta H^{{}^{\circ}}-T\Delta S^{{}^{\circ}}, \end{array}$$

(13) $$\begin{array}{c} \Delta G^{{}^{\circ}}=-\text{RT ln }K_{d},\; \end{array}$$

where *K*_*d*_ (*K*_*d*_ = *q*_*e*_/*C*_*e*_) is the partition coefficient, *R* is the gas constant (*R* = 8.314 J mol^−1^ K^−1^), and *T* is the absolute temperature of the solution (*K*).  Figure 11 shows the variation of partition coefficient *K*_*d*_ as a function of 1/*T*.

The thermodynamic parameters for the adsorption are summarized in Table 6. The positive value of Δ*H*° (46.05 kJ mol^−1^) shows that the adsorption is an endothermic process, and the positive value of Δ*S*° (253.74 J mol^−1^ K^−1^) suggests an increase in disorder at the adsorbent/solution interface during the adsorption process. The values of Δ*G*° ranged between −11.55 and −17.89 kJ mol^−1^, indicating the spontaneity and feasibility of the adsorption process, and an increase in the adsorption temperature induces a decrease of Δ*G*° value, which indicates that the adsorption process is more favorable at higher temperatures.

## 4. Conclusion

The new installation of almond crushing units in eastern Morocco generates significant amounts of the almond shell without commercial value. Indeed, the aim of this work is to exploit this waste as a low-cost adsorbent for the effluent pollutants. The obtained results for the adsorption of BG on grinding almond ASP showed that this adsorbent has an interesting adsorption capacity with a percentage of elimination of BG up to 98% for a contact time *t* < 15 min. The adsorption of BG depends on several operating parameters, such as the adsorbent dose, the initial dye concentration, the contact time, and pH. SEM analysis shows a porous and fibrous structure which explains its adsorption capacity; also, it is shown that the adsorption follows the pseudo-second-order kinetic model and the Langmuir isotherm. This result is very encouraging to valorize the almond shell by-products as low-cost bioadsorbent in the fields of wastewater treatment.

## Figures and Tables

**Table 1:** Physicochemical characteristics of Brilliant green dye.

Dye	Brilliant green
Color Index no.	42040
Chemical formula	C_27_H_34_N_2_O_4_S
Structure formula	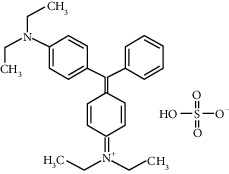
Molecular weight (g/mol)	482.63
Melting point (°C)	210
*λ* max (nm)	625

**Table 2:** Textural characteristic of almond shell powder (ASP).

ASP/BET surface area (m^2^/g)	0.19
Pores diameter BJH (Å)	798.06
Pore volume (cm³/g)	0.0003

**Table 3:** Adsorption of BG dye onto ASP: kinetic model parameters of intraparticle diffusion, pseudo-first-order, and pseudo-second-order models.

		Intraparticle diffusion			Pseudo-first-order			Pseudo-second-order		
Concentration of BG dye (mg/L)	(*q*_*e*_)_exp_	*K*_*p*_	*I*	*R*_*I*_^2^	*q*_*e*(cal)_	*K*_1_	*R*_*F*_^2^	*q*_*e*(cal)_	*K*_2_	*R*_*s*_^2^
20	19.90	0.04	19.65	0.64	15.43	0.07	0.95	20	0.28	0.99
30	29.97	0.02	29.80	0.76	20.95	0.44	0.96	30.03	0.30	0.99
40	39.91	0.20	38.51	0.92	1.54	0.06	0.94	40.16	0.08	0.99
50	49.85	0.30	47.85	0.95	2.55	0.07	0.94	50.25	0.06	0.99

**Table 4:** Isotherm constants for BG dye adsorption on almond shell powder (ASP).

Langmuir isotherm model			Freundlich isotherm model		
*q*_*m*_	*K*_*L*_	*R* ^2^	*K*_*F*_	*n*	*R* ^2^
58.13	43.00	0.993	85.34	3.54	0.97

**Table 5:** Example of data from studies of different adsorbents used for removal of different dyes.

Adsorbent	Adsorbate	Amount of adsorption (mg/g)	Kinetic model	Adsorption isotherm	Reference
ASP	BG dye	58.13	Pseudo-second-order	Langmuir	Present study
Bambusa Tulda-Na_2_CO_3_	BG dye	41.67	Pseudo-second-order	Langmuir	[37]
Bambusa Tulda-HCL	BG dye	31.25	Pseudo-second-order	Langmuir	[37]
Areca nut husk	BG dye	18.21	Pseudo-second-order	Langmuir	[45]
*Pinus roxburghii* leaves	BG dye	71.42	Pseudo-second-order	Langmuir	[46]
Bagasse fly ash	BG dye	133.33	Pseudo-second-order	Langmuir	[47]
Luffa cylindrical sponge	BG dye	18.20	Pseudo-second-order	Langmuir	[48]
Almond shell	Methylene blue	833.33	Pseudo-second-order	Langmuir	[49]
Almond shell	Crystal violet	625	Pseudo-second-order	Langmuir	[49]
Almond shell	Rhodamine 6G	32.6	Pseudo-second-order	Langmuir	[50]
Almond shell	2-Picoline	288.57	Pseudo-second-order	Langmuir	[44]
Walnut shells powder	Methylene blue	178.9	Pseudo-second-order	Langmuir	[10]
Walnut sawdust	Methylene blue	59.17	Pseudo-second-order	Langmuir	[51]
Apricot shell	Bisphenol A	51.91	Pseudo-second-order	Langmuir	[52]
Apricot shell	Atrazine	56.91	Pseudo-second-order	Freundlich	[52]
Pineapple leaf powder	Crystal violet	78.22	Pseudo-second-order	Langmuir	[53]	
Punica granatum shell	Crystal violet	50.21	Pseudo-second-order	Langmuir	[54]	
Tea waste	Astrazon blue FGRL	263.16	Not cited	Freundlich	[55]	

**Table 6:** Thermodynamic parameters of the adsorption process of BG dye onto ASP at various temperatures.

Δ*H* (kJ·mol^−1^)	Δ*S* (J·mol^−1^·K^−1^)	Δ*G*° (kJ·mol^−1^) at tested temperatures			
		20°C	30°C	40°C	50°C
64.059	253.740	−11.556	−12.824	−15.362	−17.899

## Data Availability

The data used to support the findings of this study are available from the corresponding author upon request.
